# Supplementary figures and images for: Disrupting the ciliary gradient of active Arl3 affects rod photoreceptor nuclear migration
Source: eLife. 2023 Jan 4;12:e80533. doi: 10.7554/eLife.80533 (PMC9831603; doi:10.7554/eLife.80533)

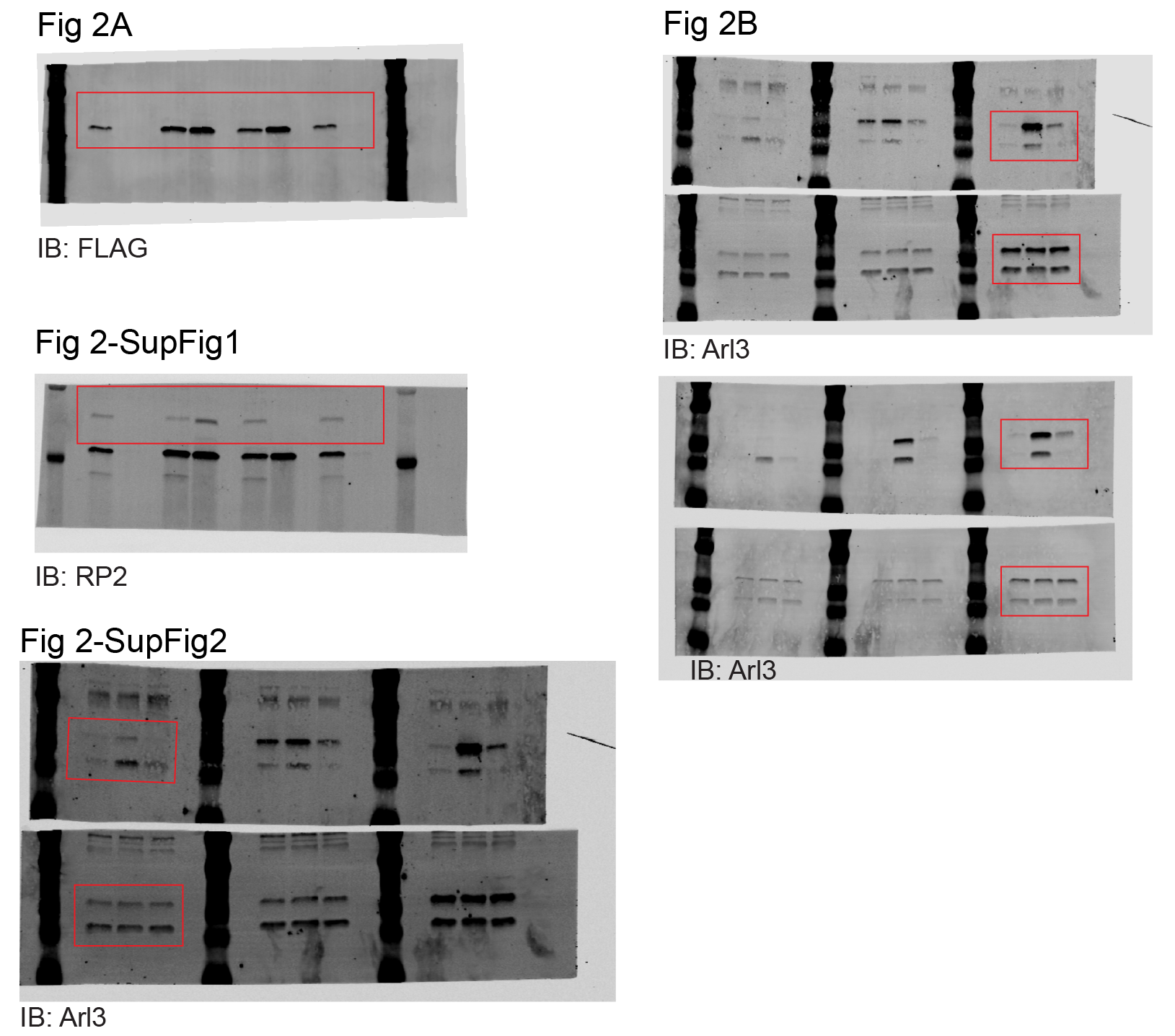

Supplement: Figure 2—source data 1. [file elife-80533-fig2-data1.zip › UncroppedWB_Fig 2.png]

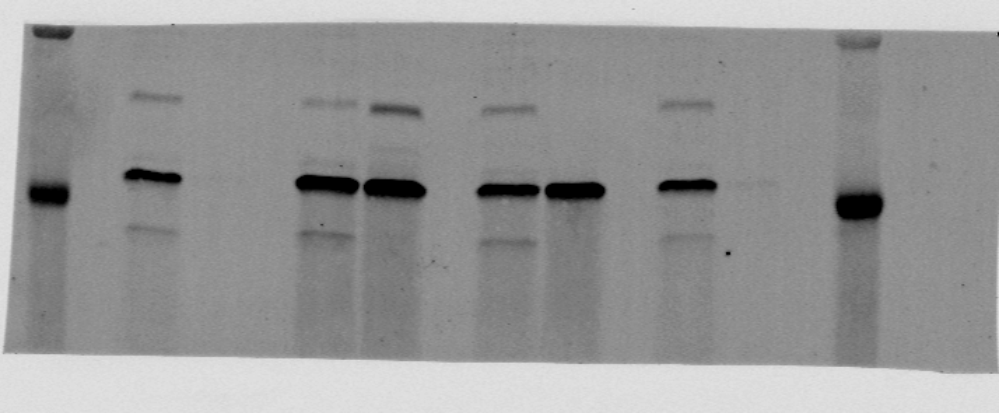

Supplement: Figure 2—source data 1. [file elife-80533-fig2-data1.zip › Fig 2sup2.png]

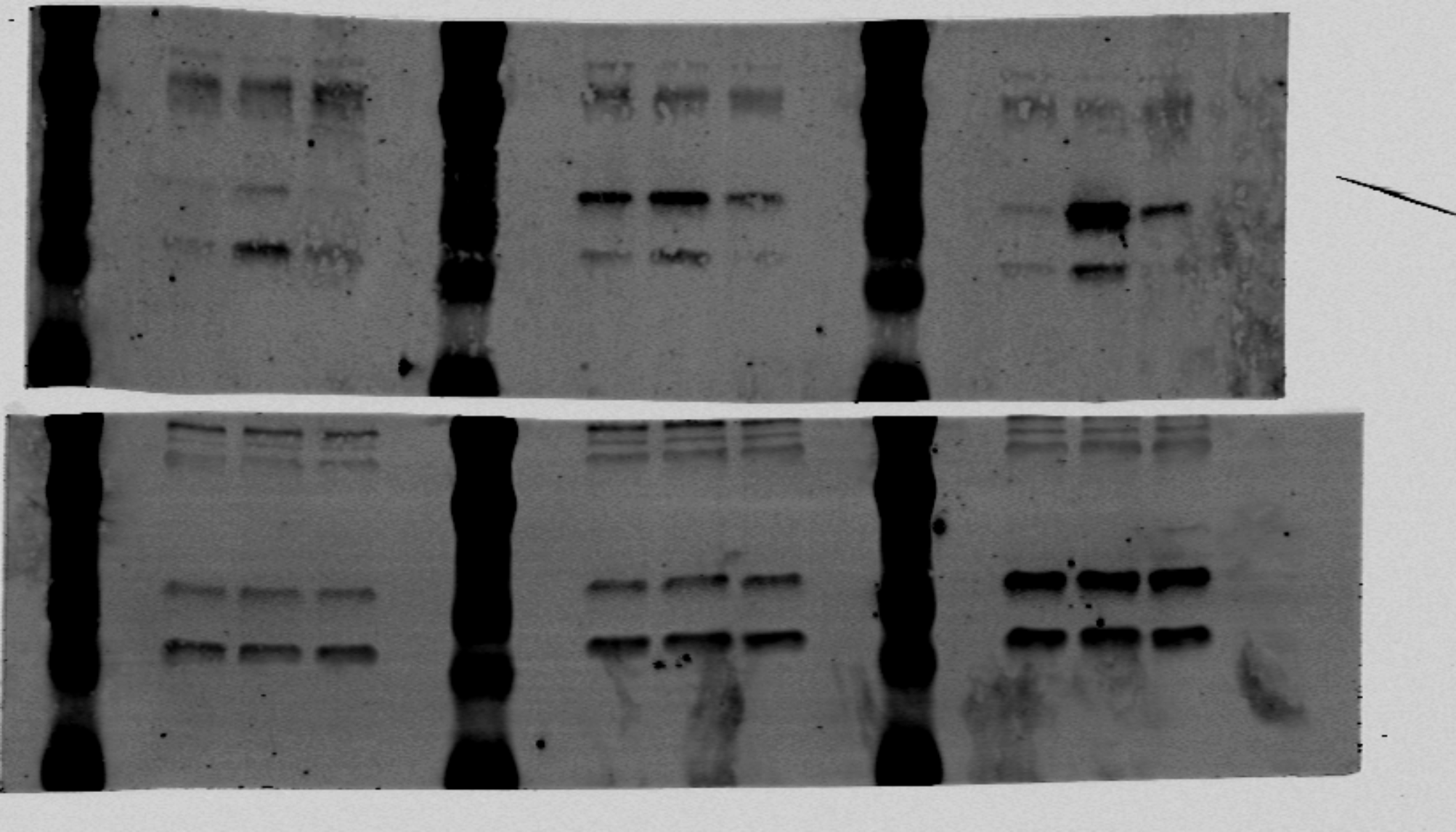

Supplement: Figure 2—source data 1. [file elife-80533-fig2-data1.zip › Fig 2sup1-Arl3.tif]

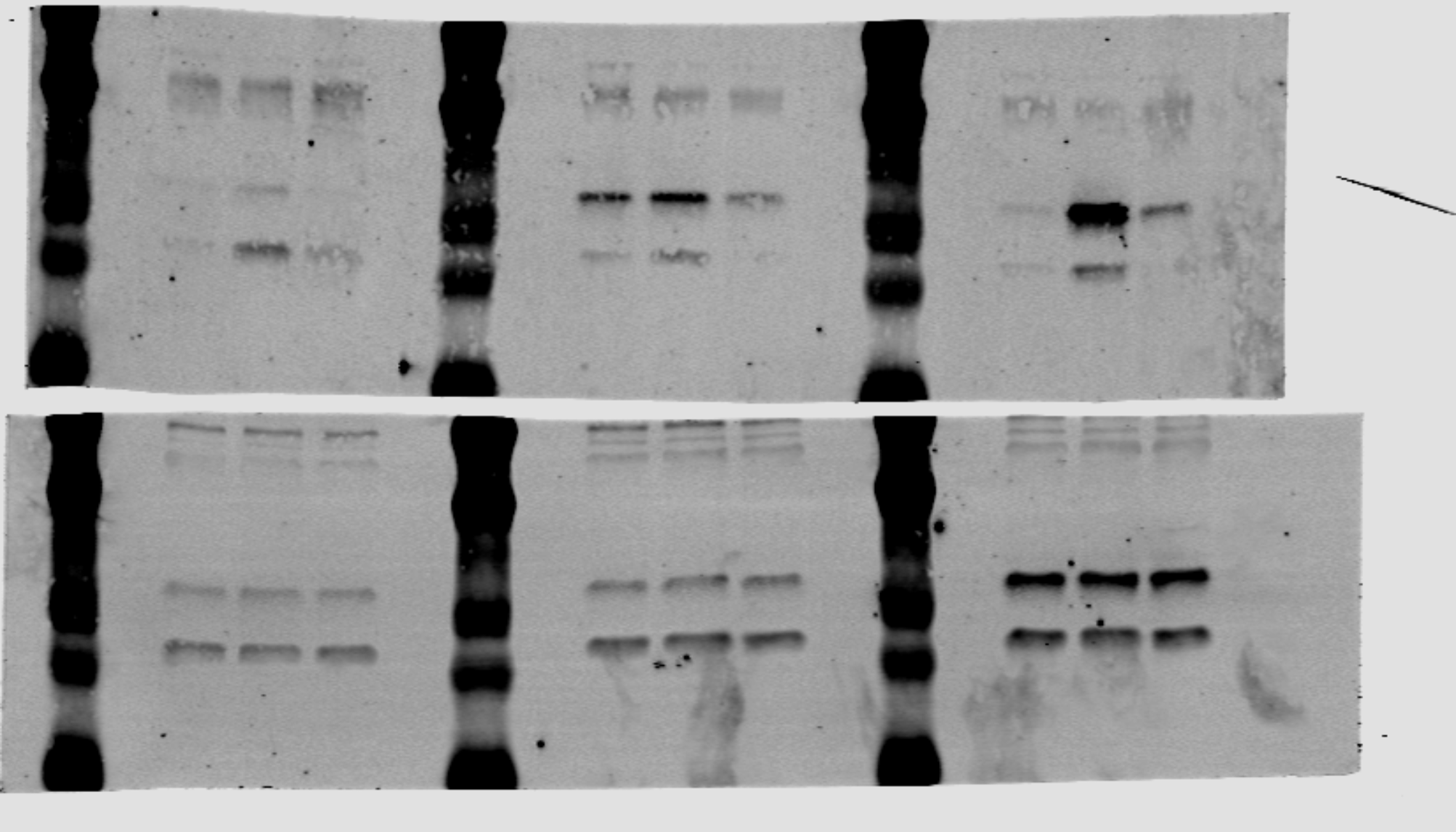

Supplement: Figure 2—source data 1. [file elife-80533-fig2-data1.zip › Fig 2B Y90C.tif]

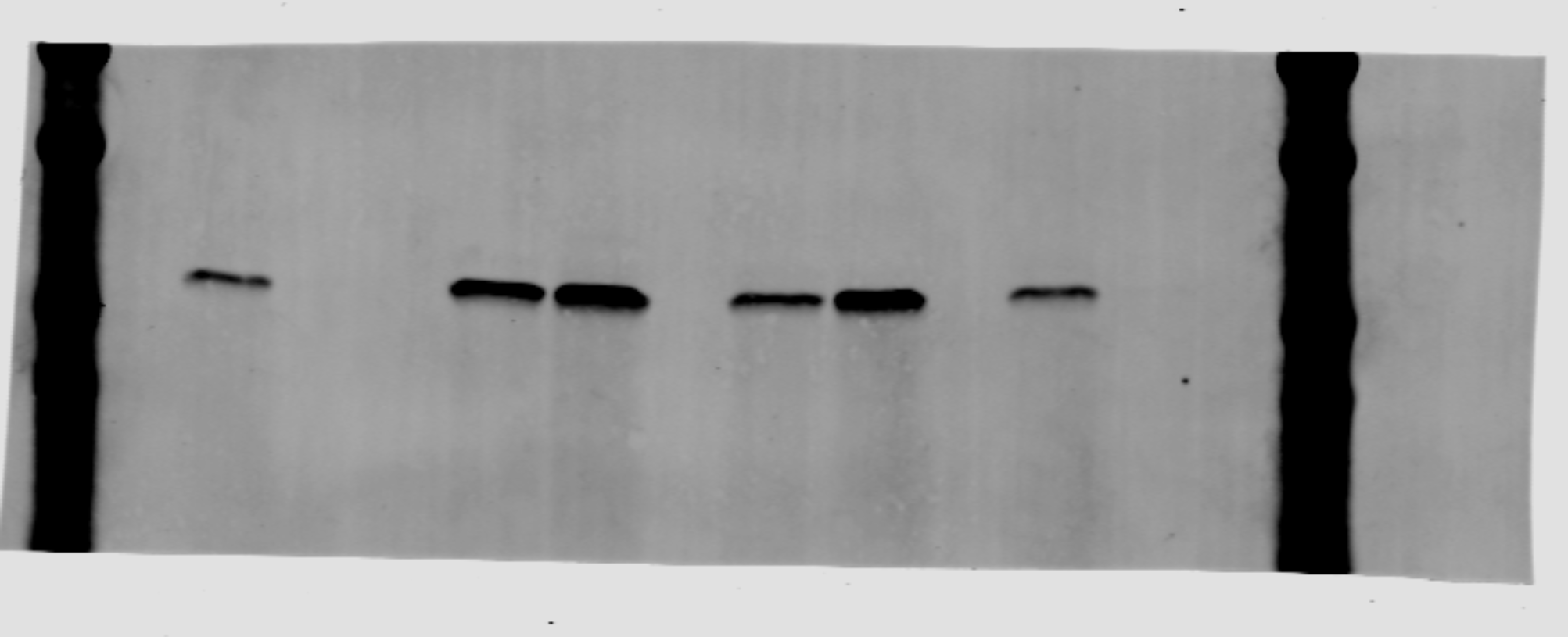

Supplement: Figure 2—source data 1. [file elife-80533-fig2-data1.zip › Fig 2A.tif]

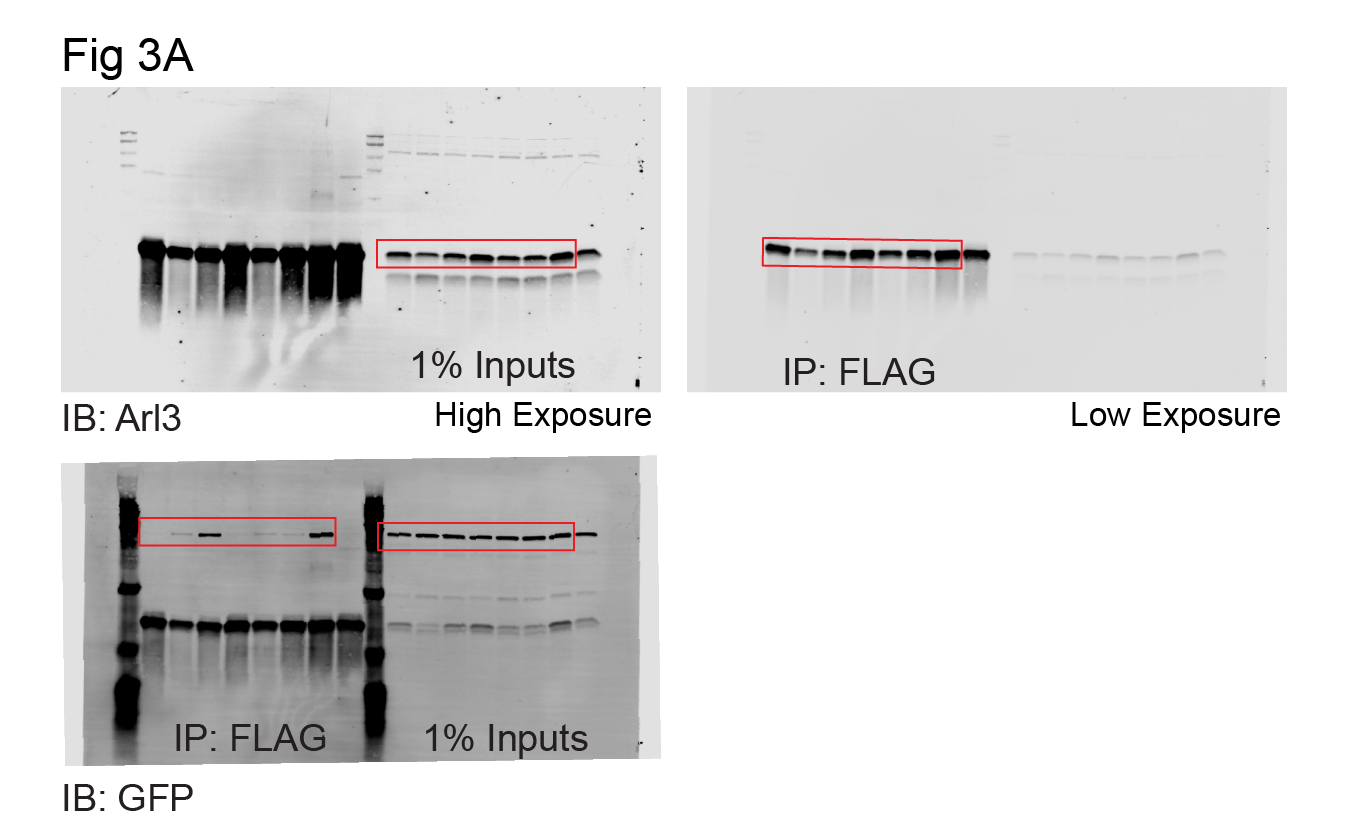

Supplement: Figure 3—source data 1. [file elife-80533-fig3-data1.zip › UncroppedWB_Fig 3.png]

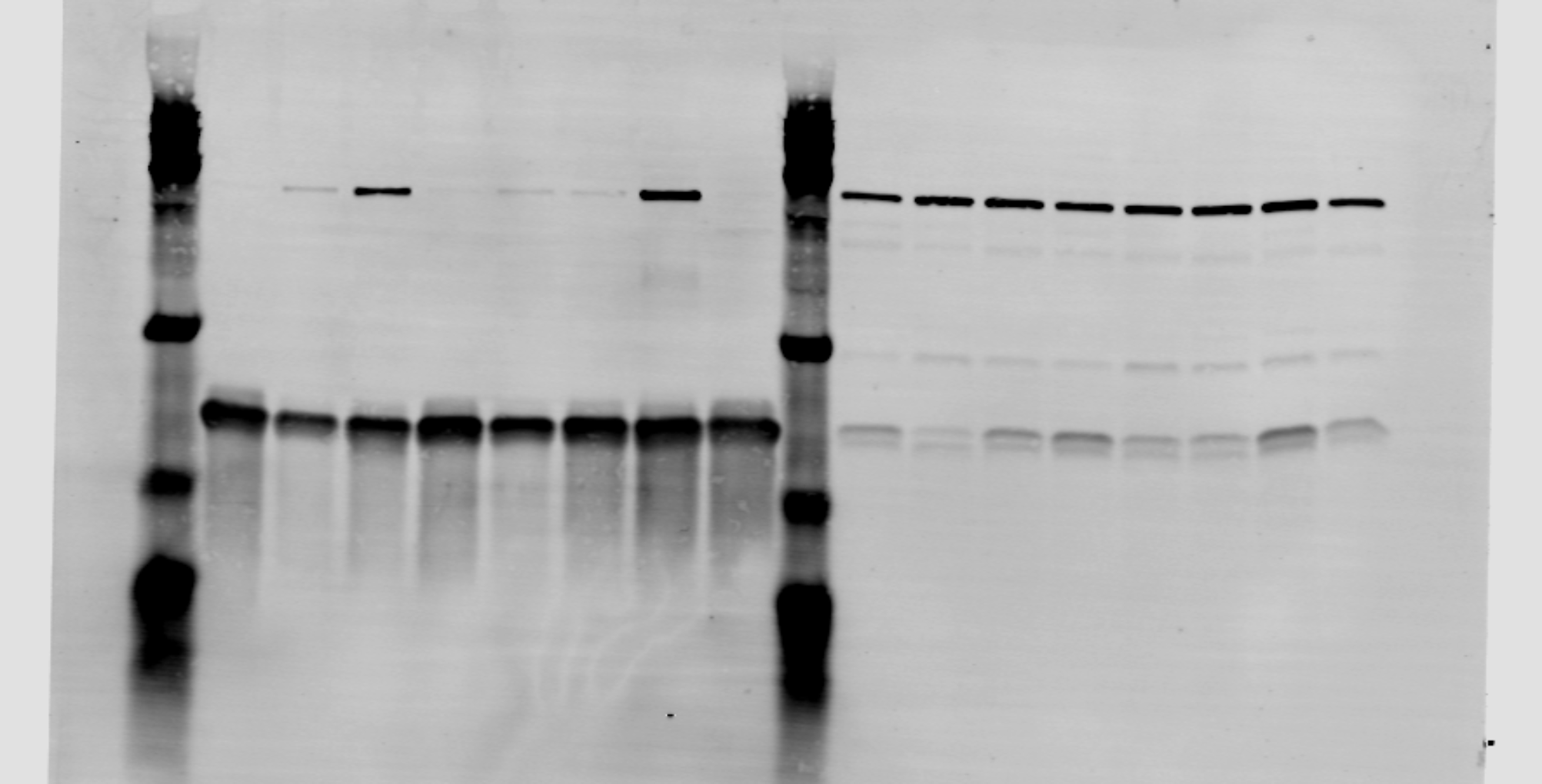

Supplement: Figure 3—source data 1. [file elife-80533-fig3-data1.zip › Fig 3A-Arl13b IP and inputs.png]

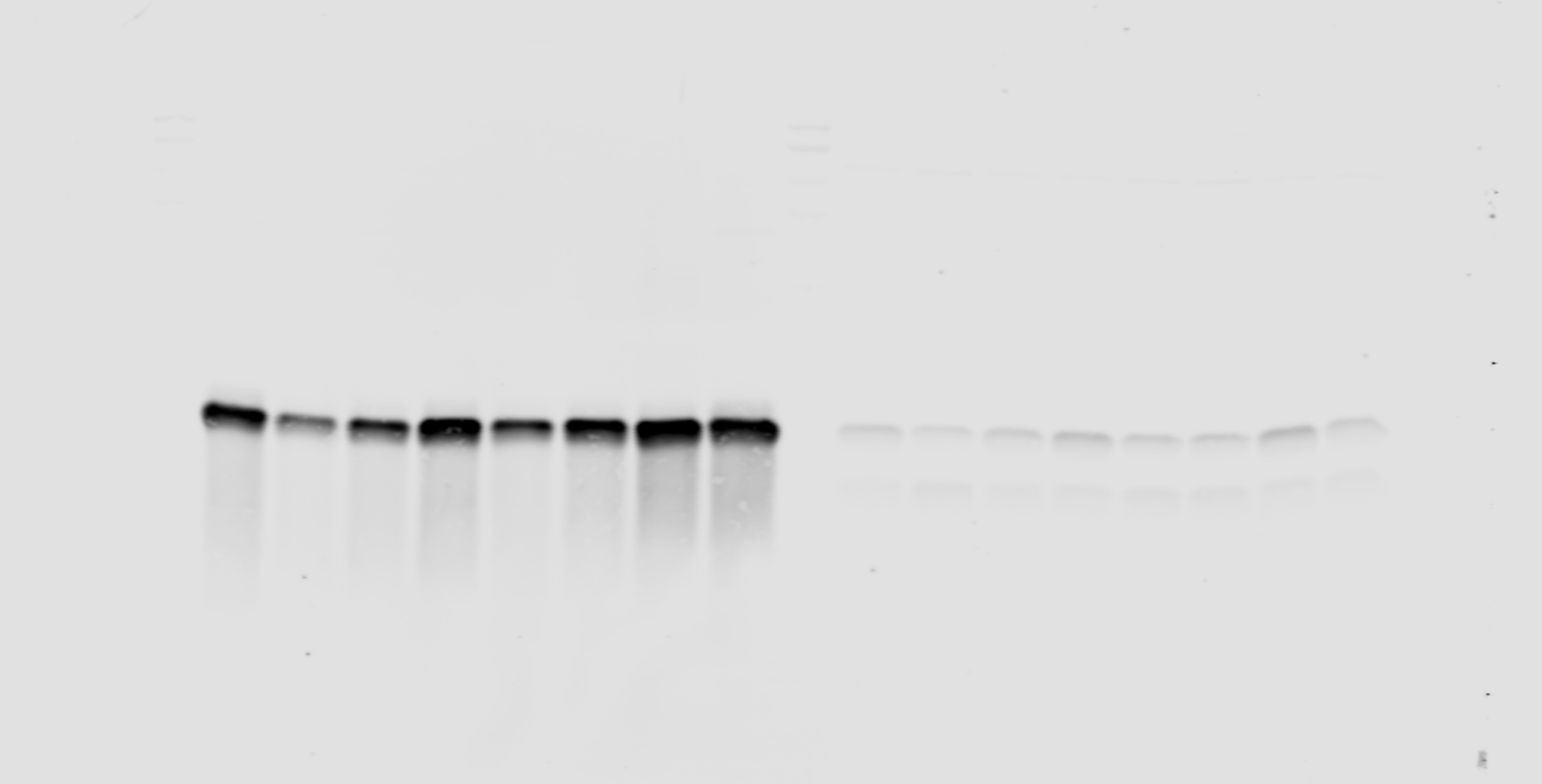

Supplement: Figure 3—source data 1. [file elife-80533-fig3-data1.zip › Fig 3A Arl3 ip.png]

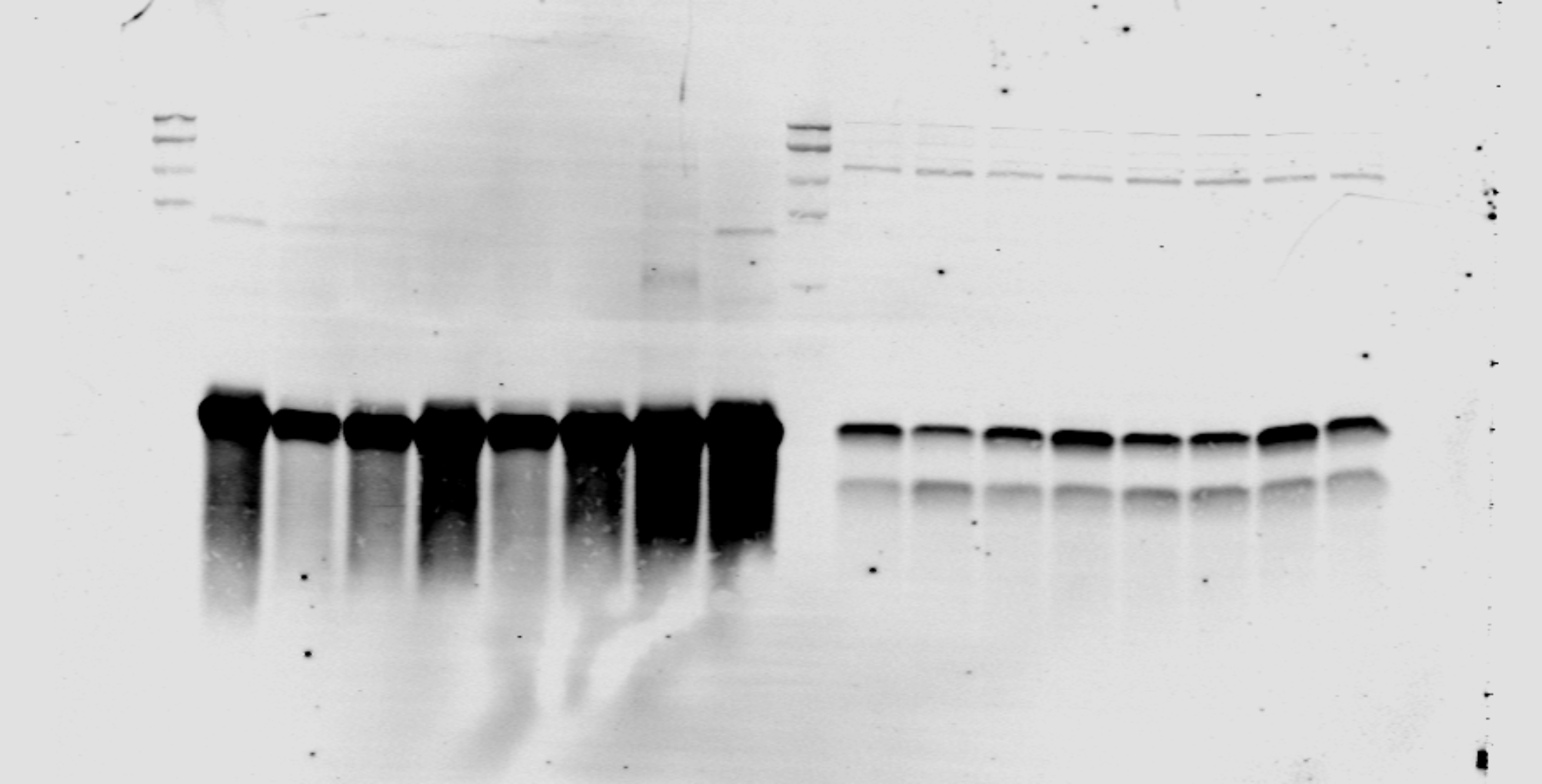

Supplement: Figure 3—source data 1. [file elife-80533-fig3-data1.zip › Fig 3A Arl3 input.png]

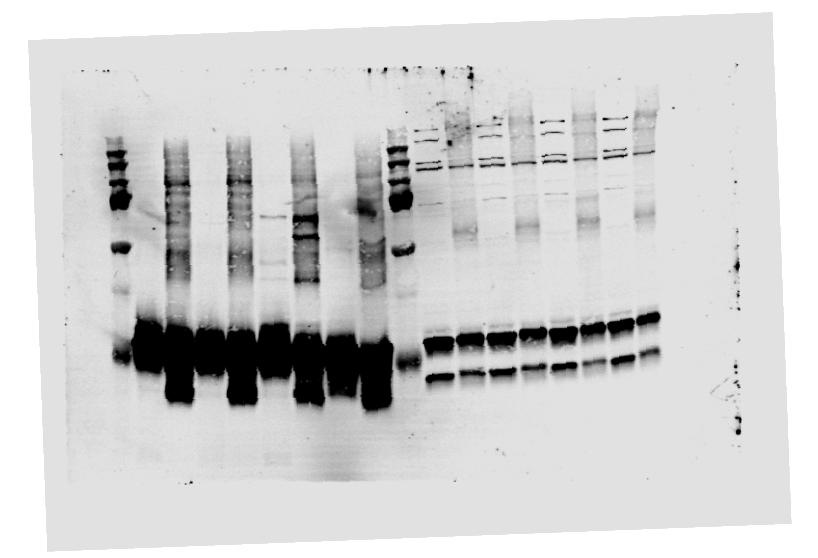

Supplement: Figure 4—source data 2. [file elife-80533-fig4-data2.zip › Fig 4A.png]

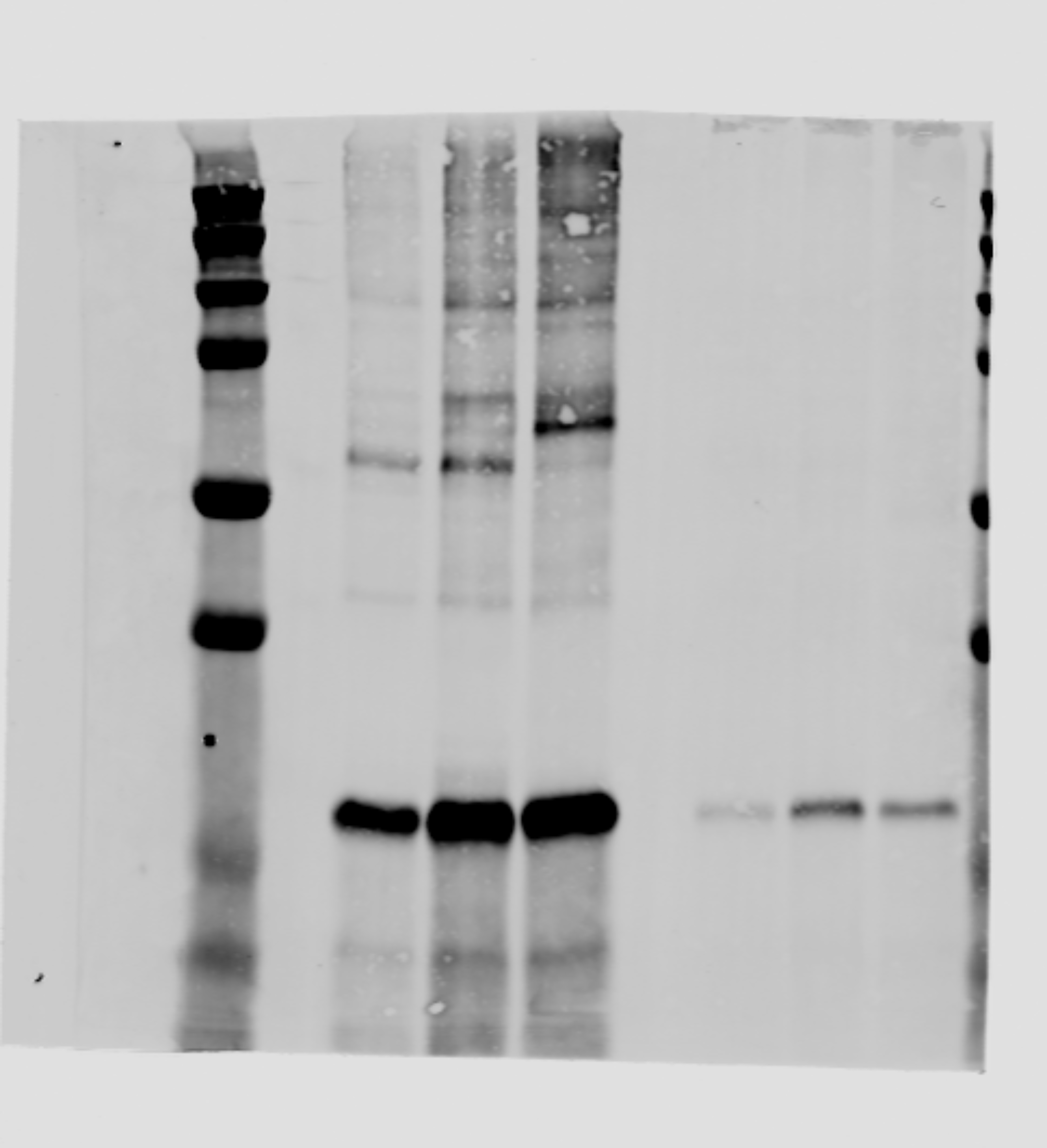

Supplement: Figure 4—source data 2. [file elife-80533-fig4-data2.zip › Fig 4C FLAG.png]

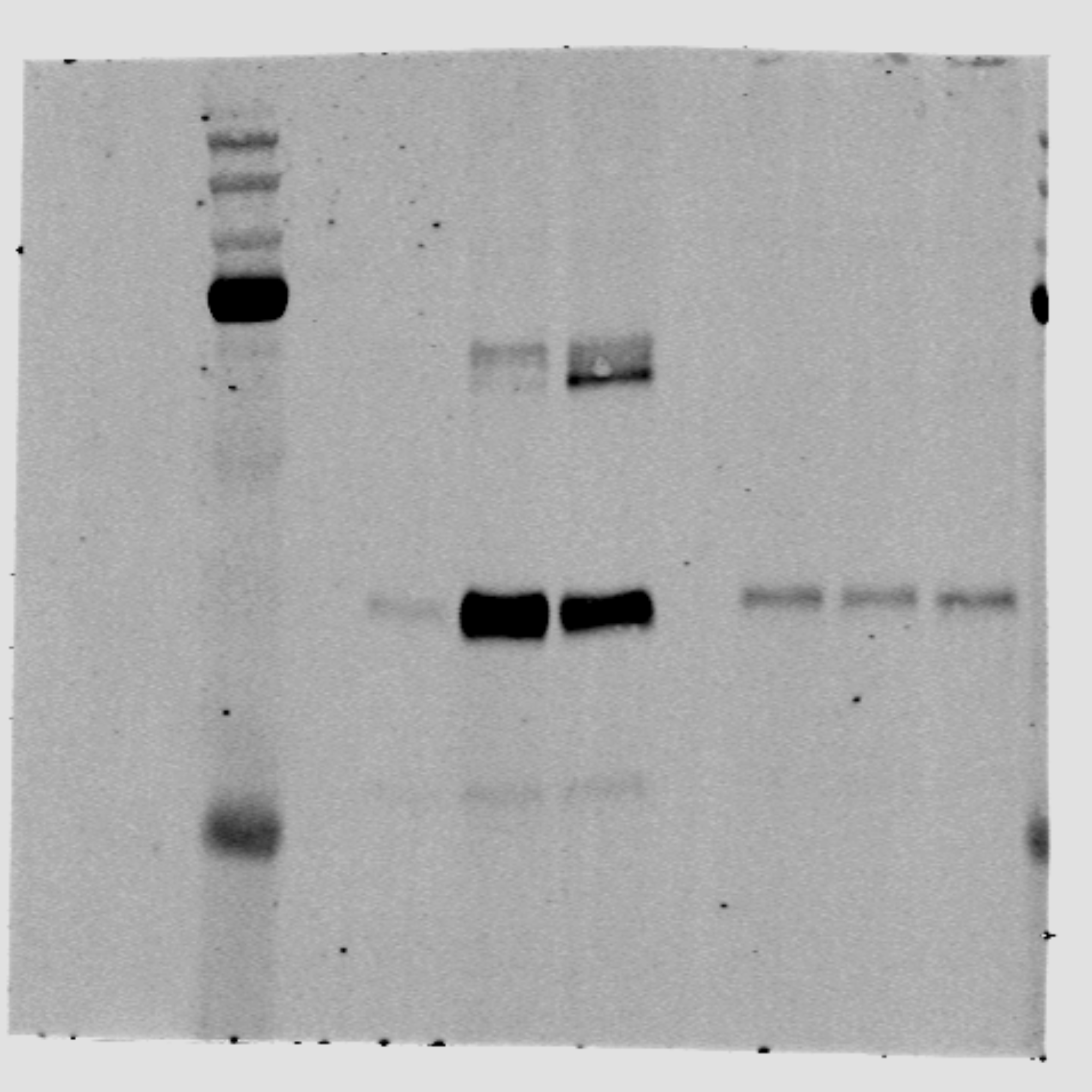

Supplement: Figure 4—source data 2. [file elife-80533-fig4-data2.zip › Fig 4C RP2.png]

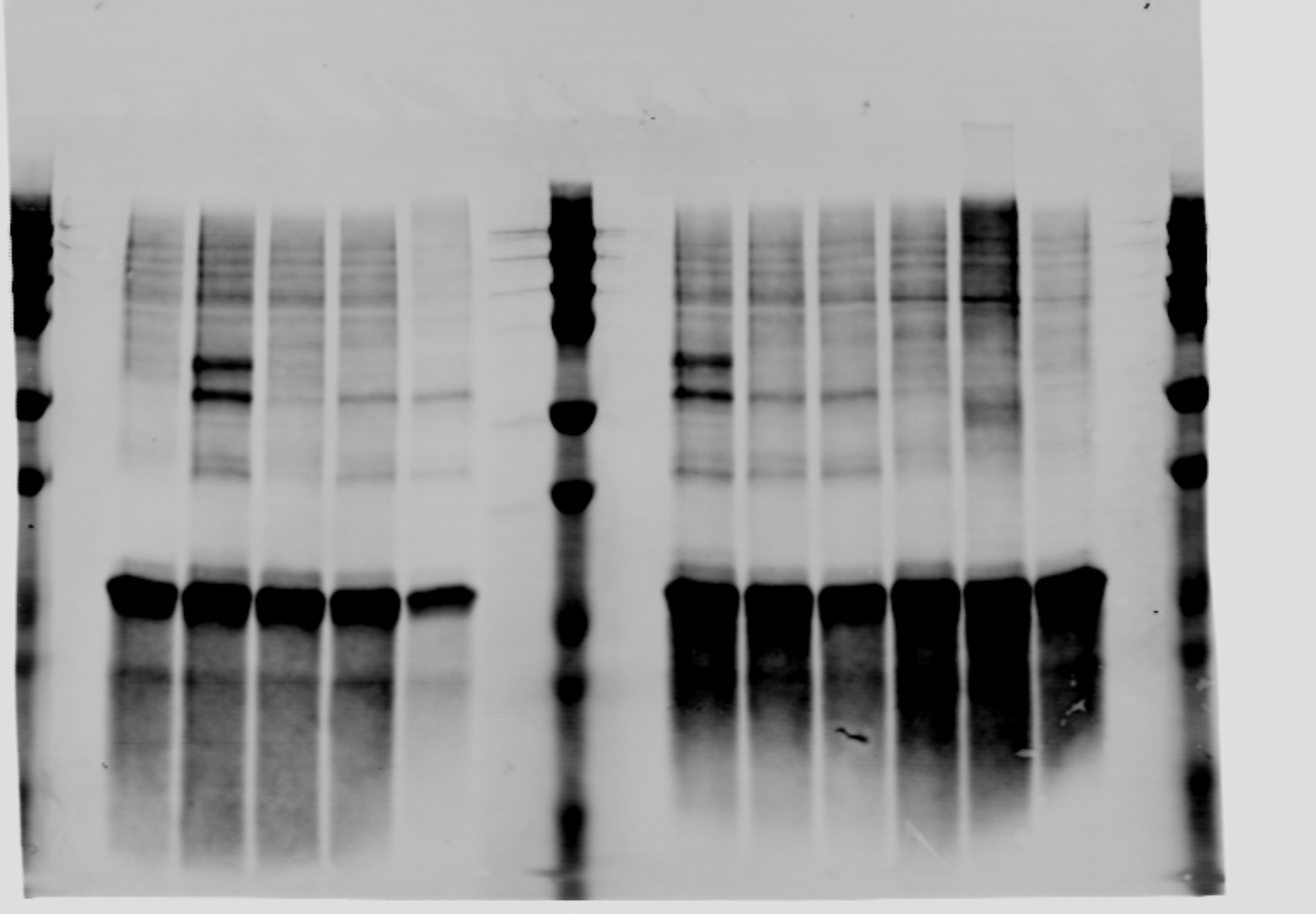

Supplement: Figure 4—source data 2. [file elife-80533-fig4-data2.zip › Fig 4D (left) and Fig 3F (right).png]

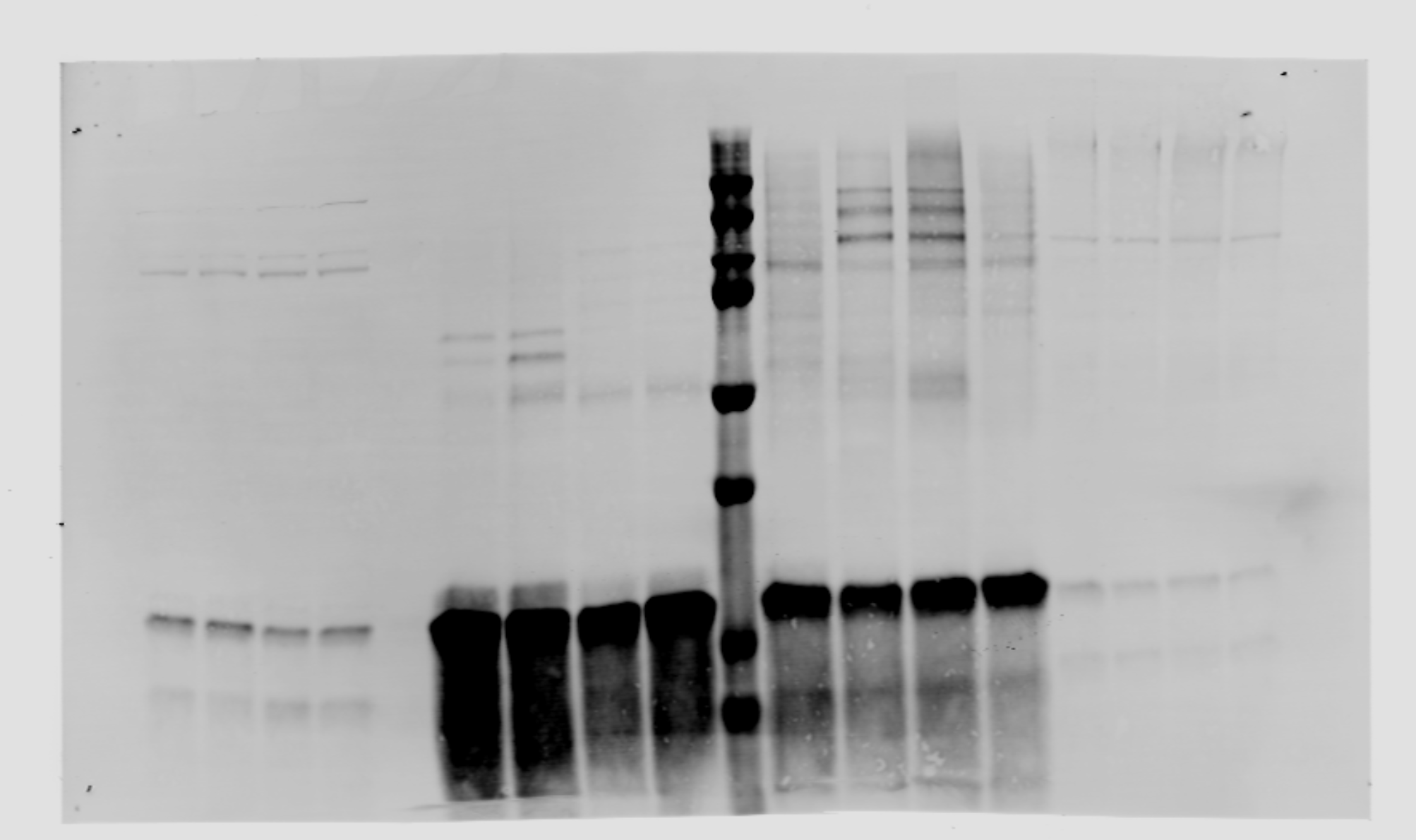

Supplement: Figure 4—source data 2. [file elife-80533-fig4-data2.zip › Fig 4G Arl3.tif]

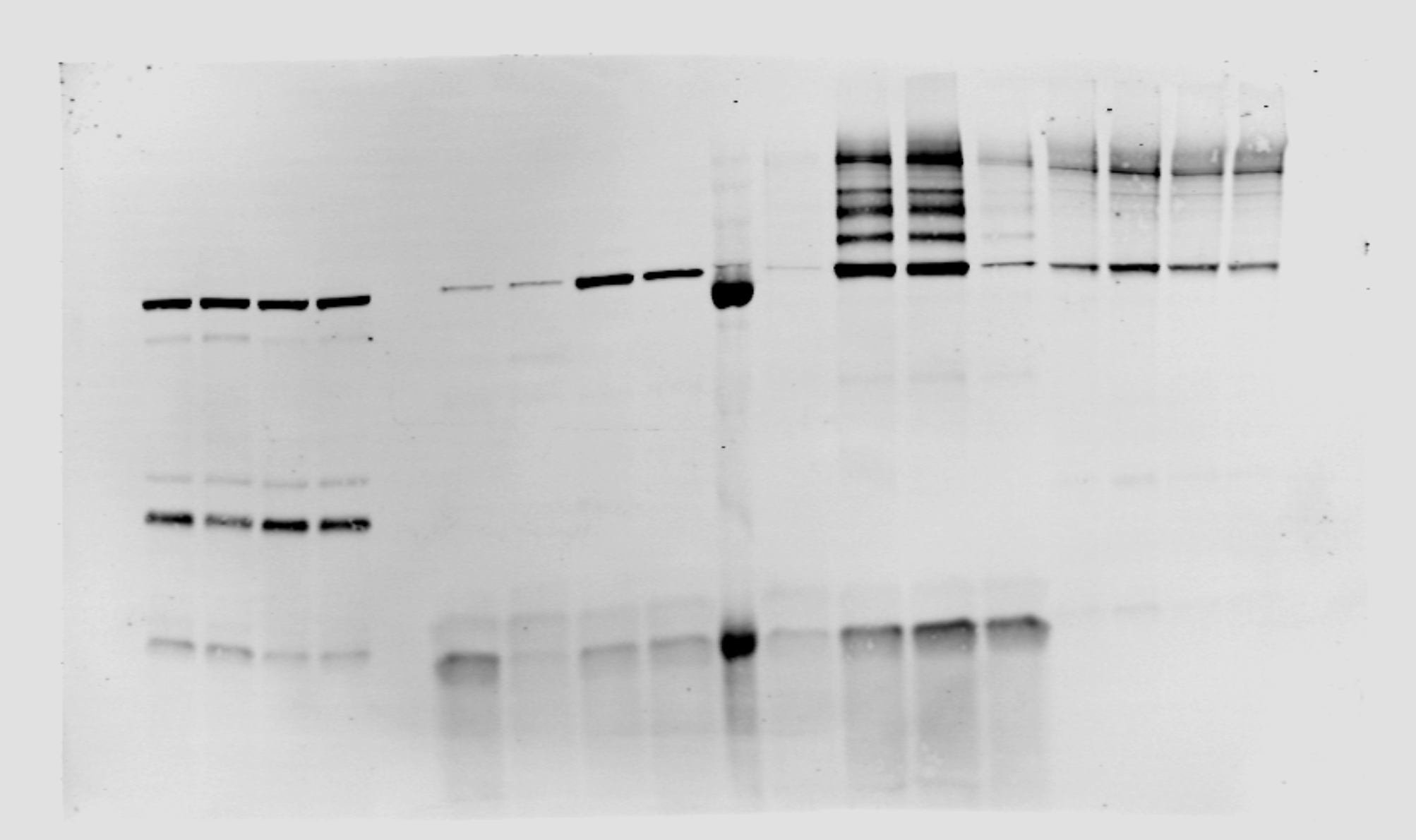

Supplement: Figure 4—source data 2. [file elife-80533-fig4-data2.zip › Fig 4G GFP.tif]

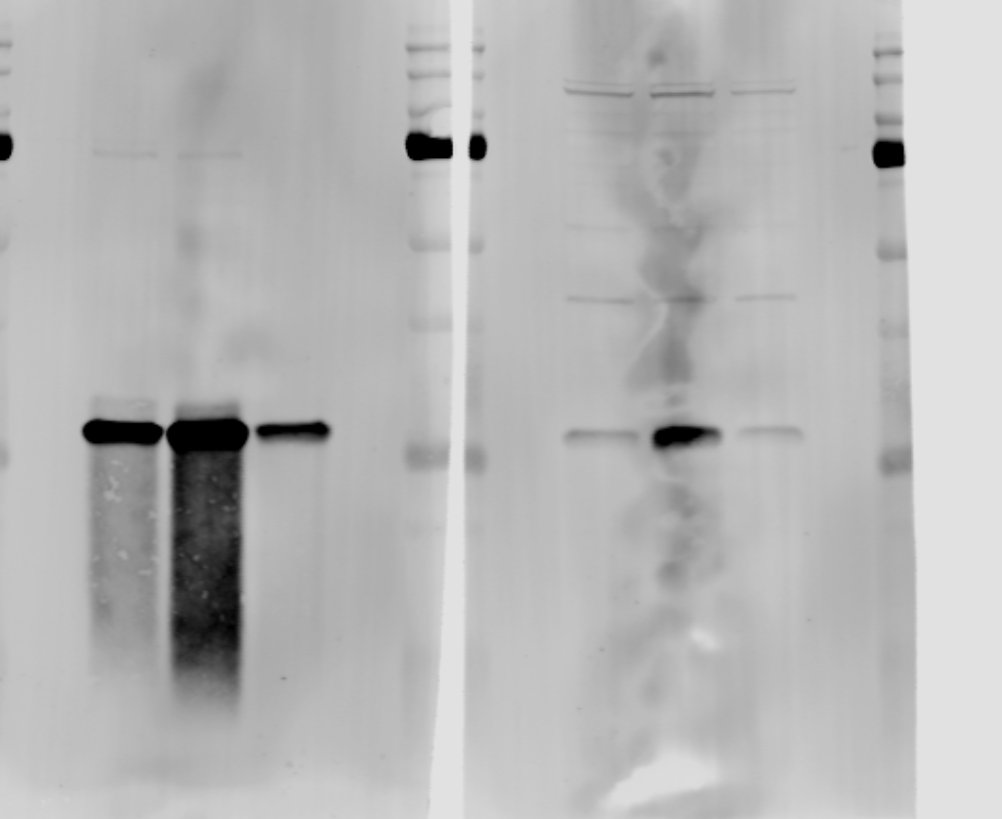

Supplement: Figure 4—source data 2. [file elife-80533-fig4-data2.zip › Fig 4sup1 FLAG.png]

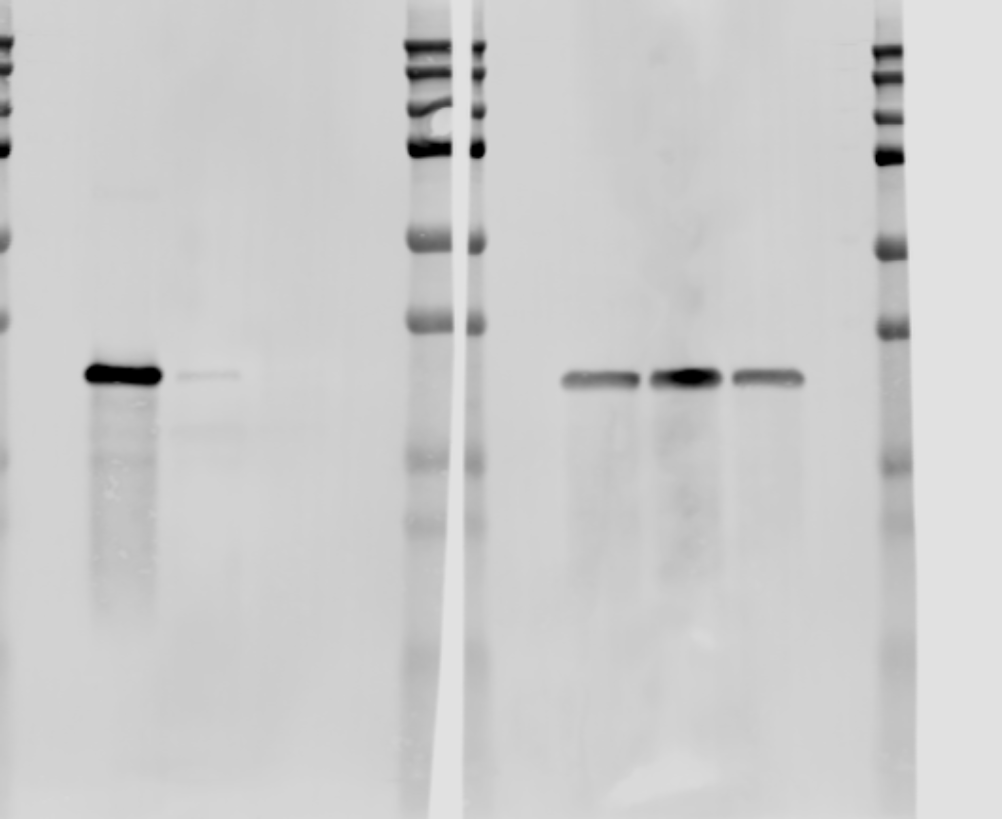

Supplement: Figure 4—source data 2. [file elife-80533-fig4-data2.zip › Fig 4sup1 MYC.png]

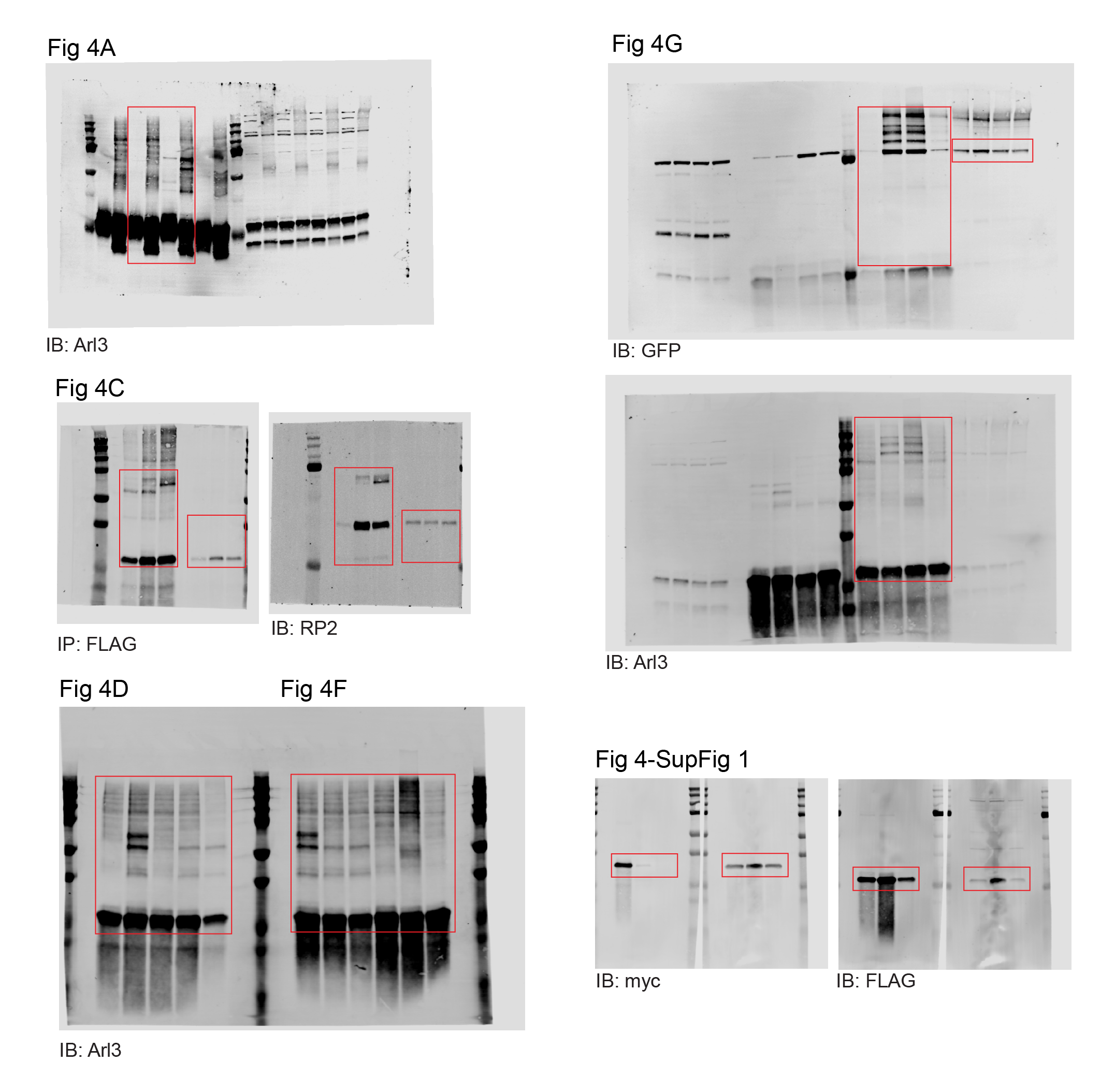

Supplement: Figure 4—source data 2. [file elife-80533-fig4-data2.zip › UncroppedWB_Fig 4.png]

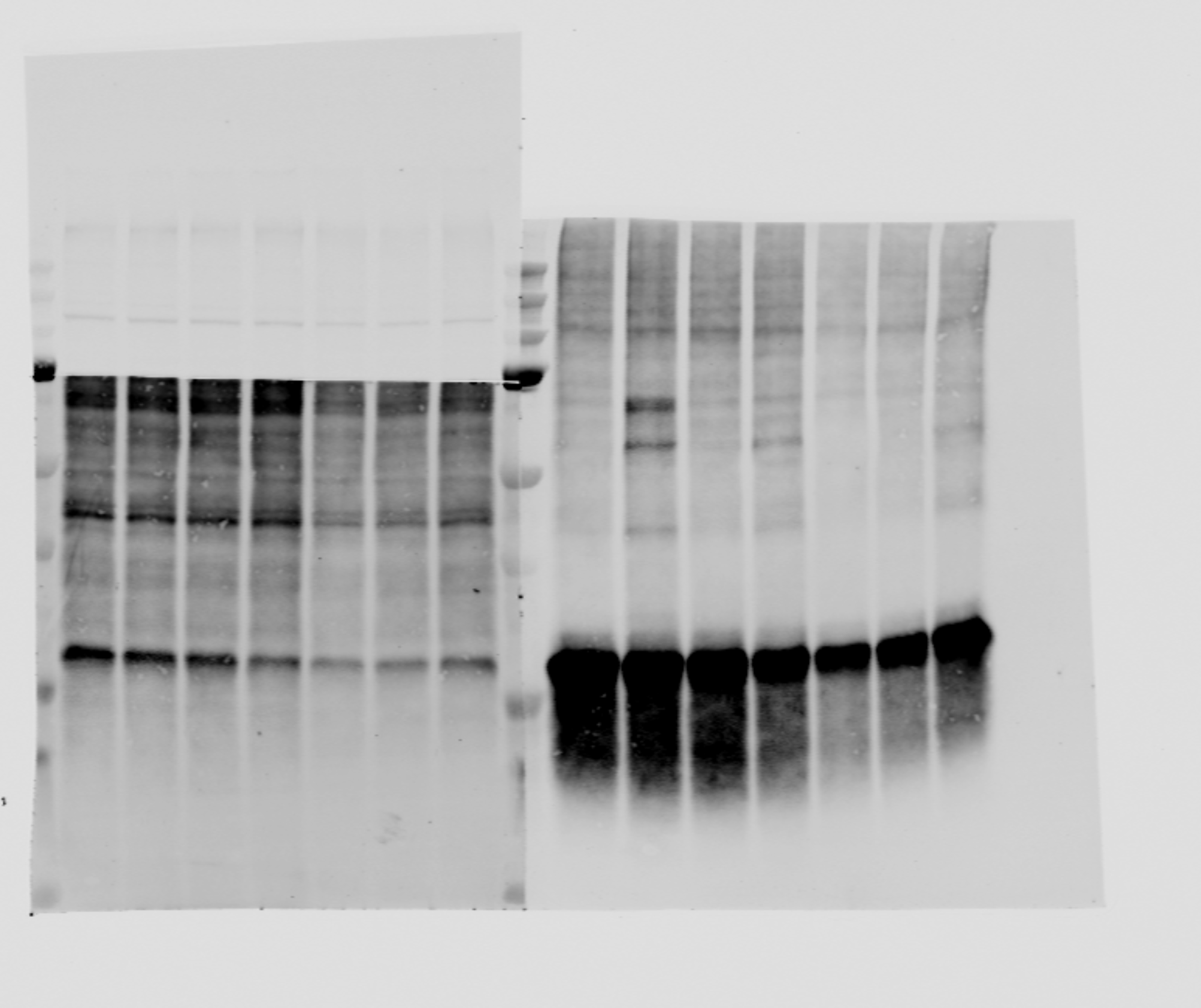

Supplement: Figure 5—source data 1. [file elife-80533-fig5-data1.zip › Fig 5A Arl3 input (top) and FLAG input (bottom) and Arl3 IP (right).png]

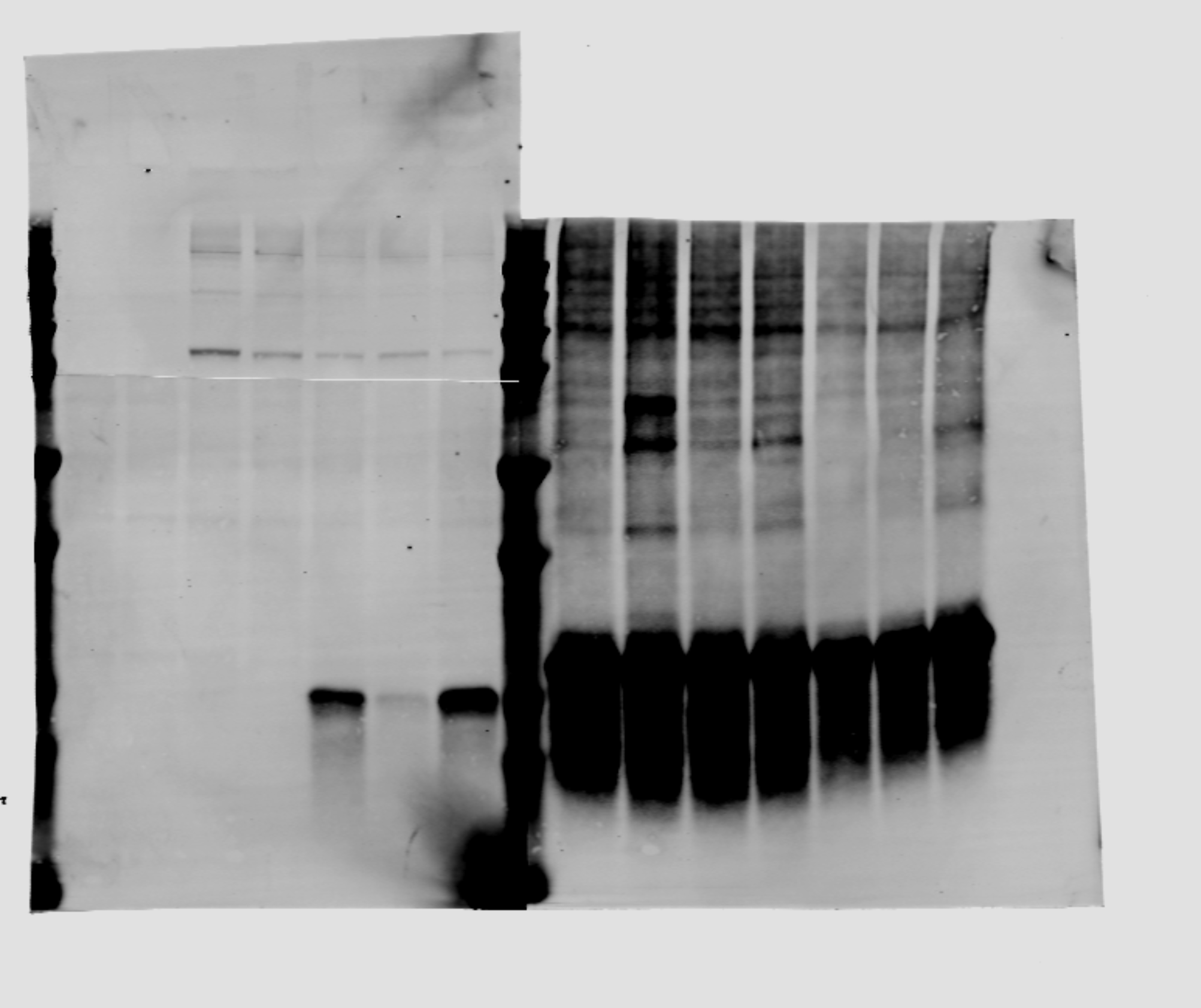

Supplement: Figure 5—source data 1. [file elife-80533-fig5-data1.zip › Fig 5A GFP input (top) and MYC input (bottom) and FLAG IP (right).png]

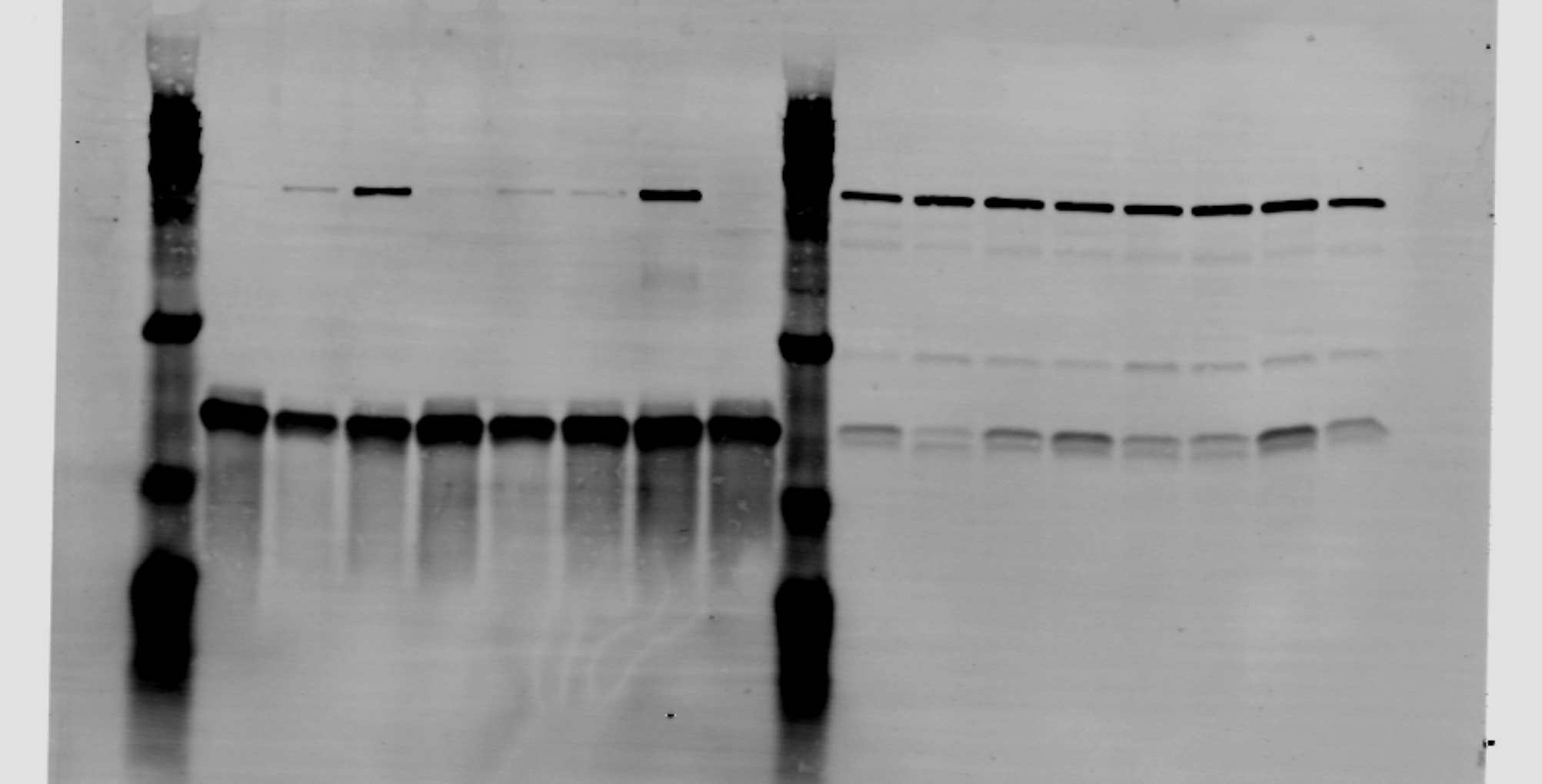

Supplement: Figure 5—source data 1. [file elife-80533-fig5-data1.zip › Fig 5A GFP.png]

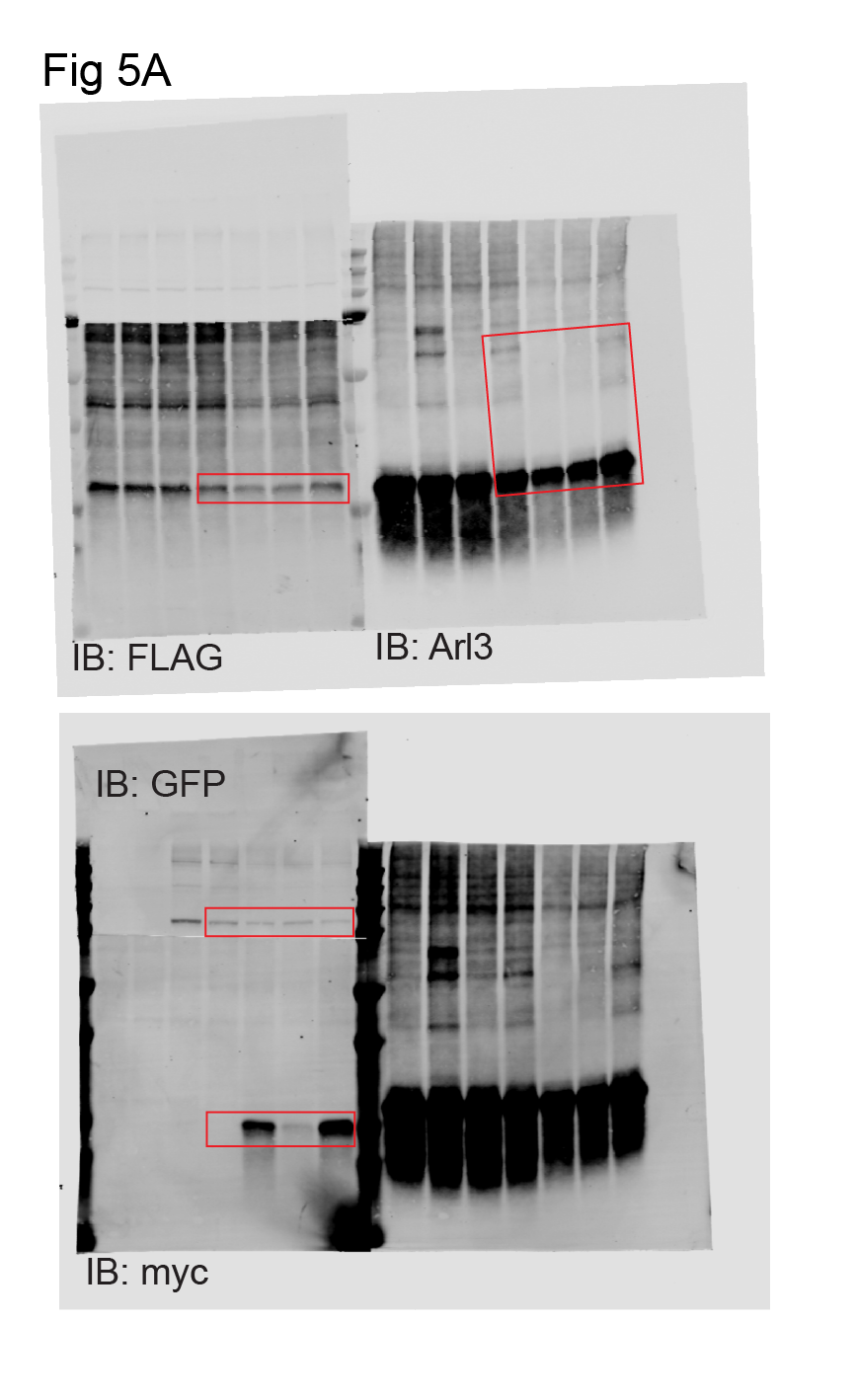

Supplement: Figure 5—source data 1. [file elife-80533-fig5-data1.zip › UncroppedWB_Fig 5.png]

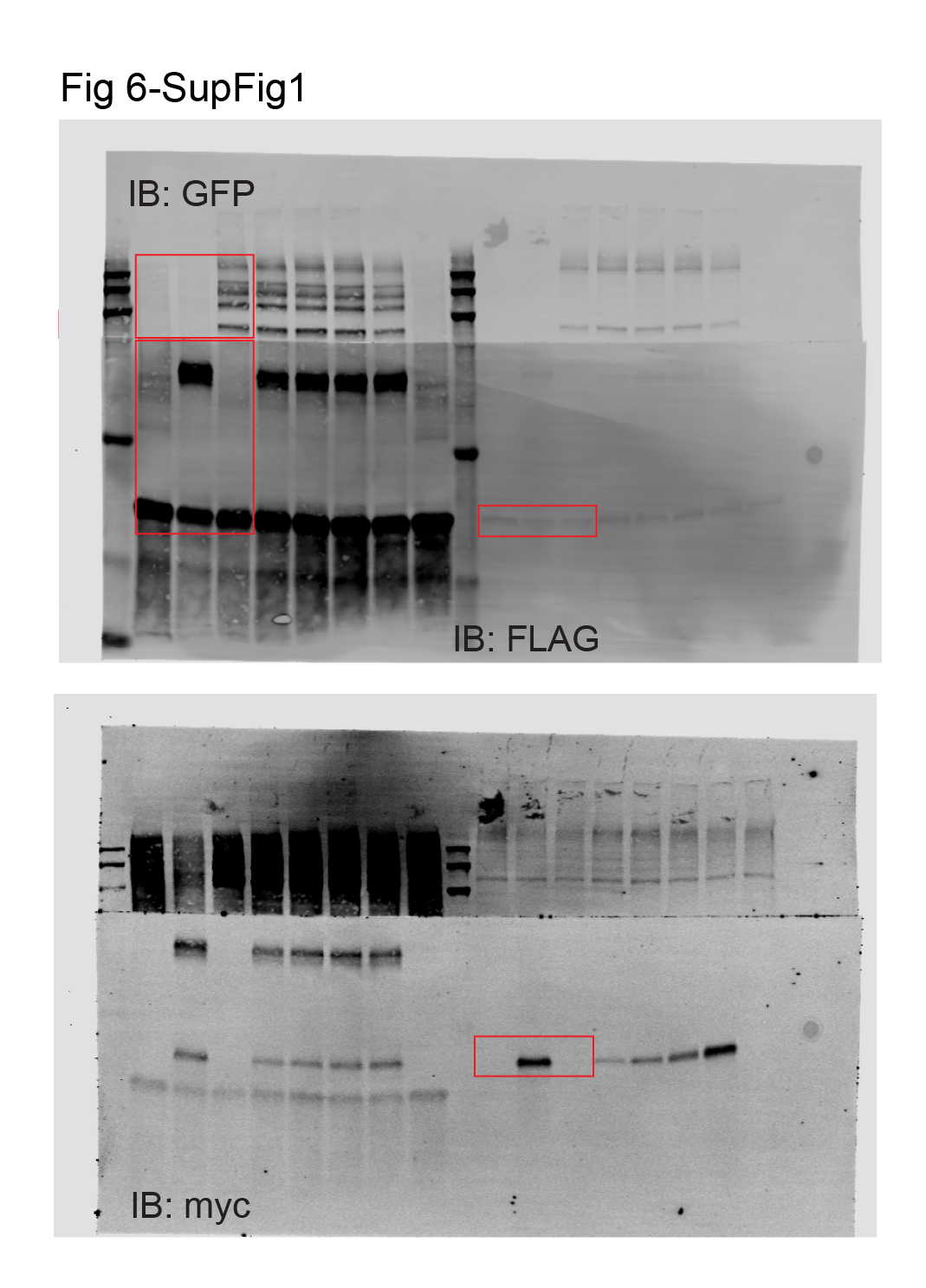

Supplement: Figure 6—source data 1. [file elife-80533-fig6-data1.zip › UncroppedWB_Fig 6.png]

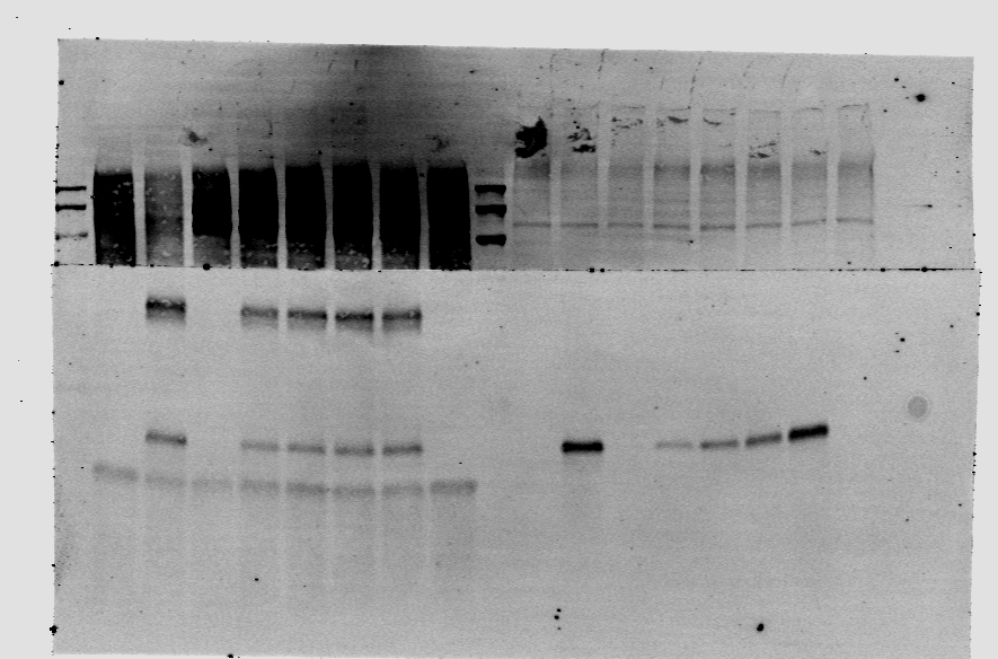

Supplement: Figure 6—source data 1. [file elife-80533-fig6-data1.zip › Fig 6sup1 MYC (bottom).png]

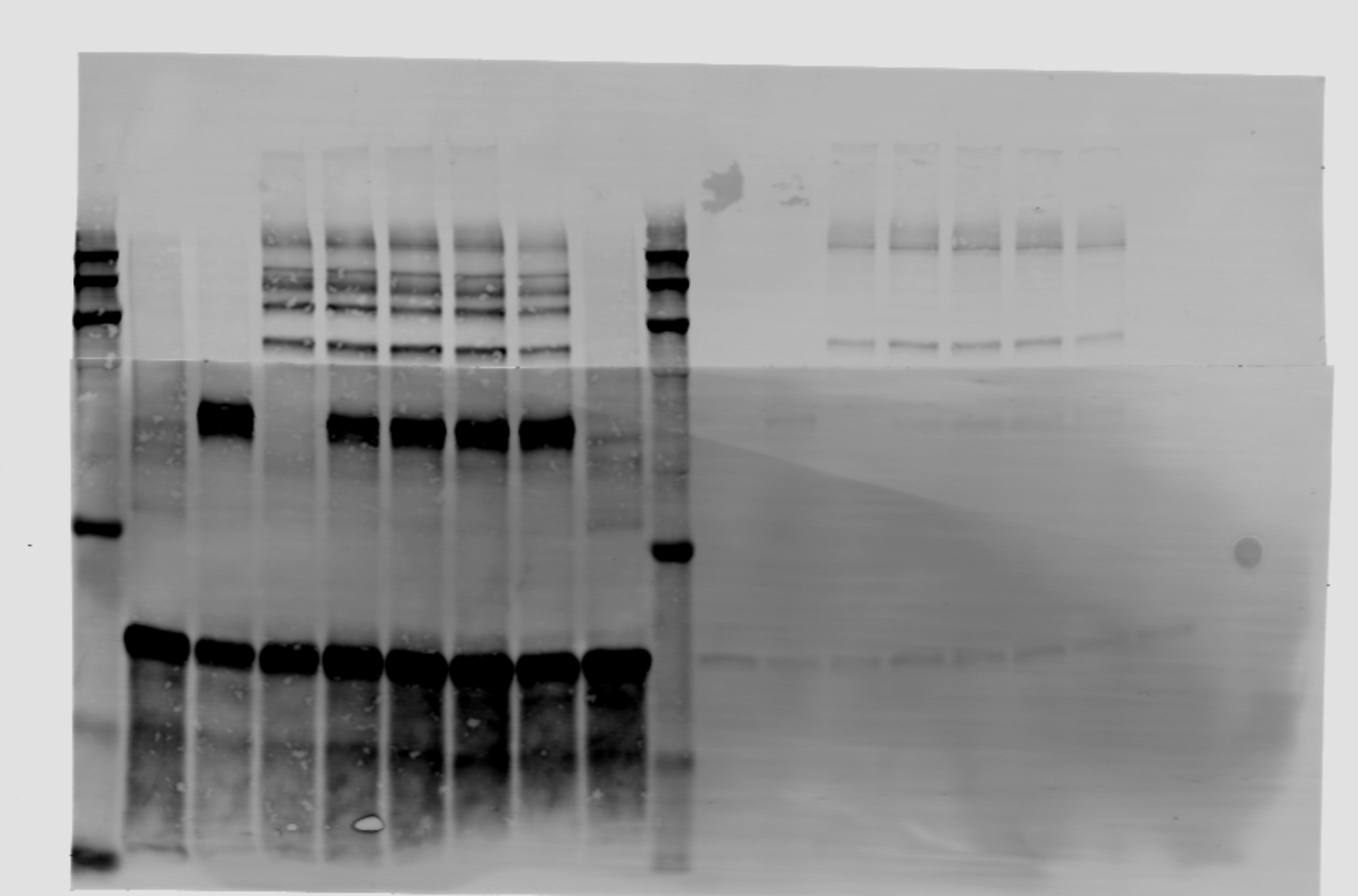

Supplement: Figure 6—source data 1. [file elife-80533-fig6-data1.zip › Fig 6sup1 GFP (top) and FLAG (bottom).png]

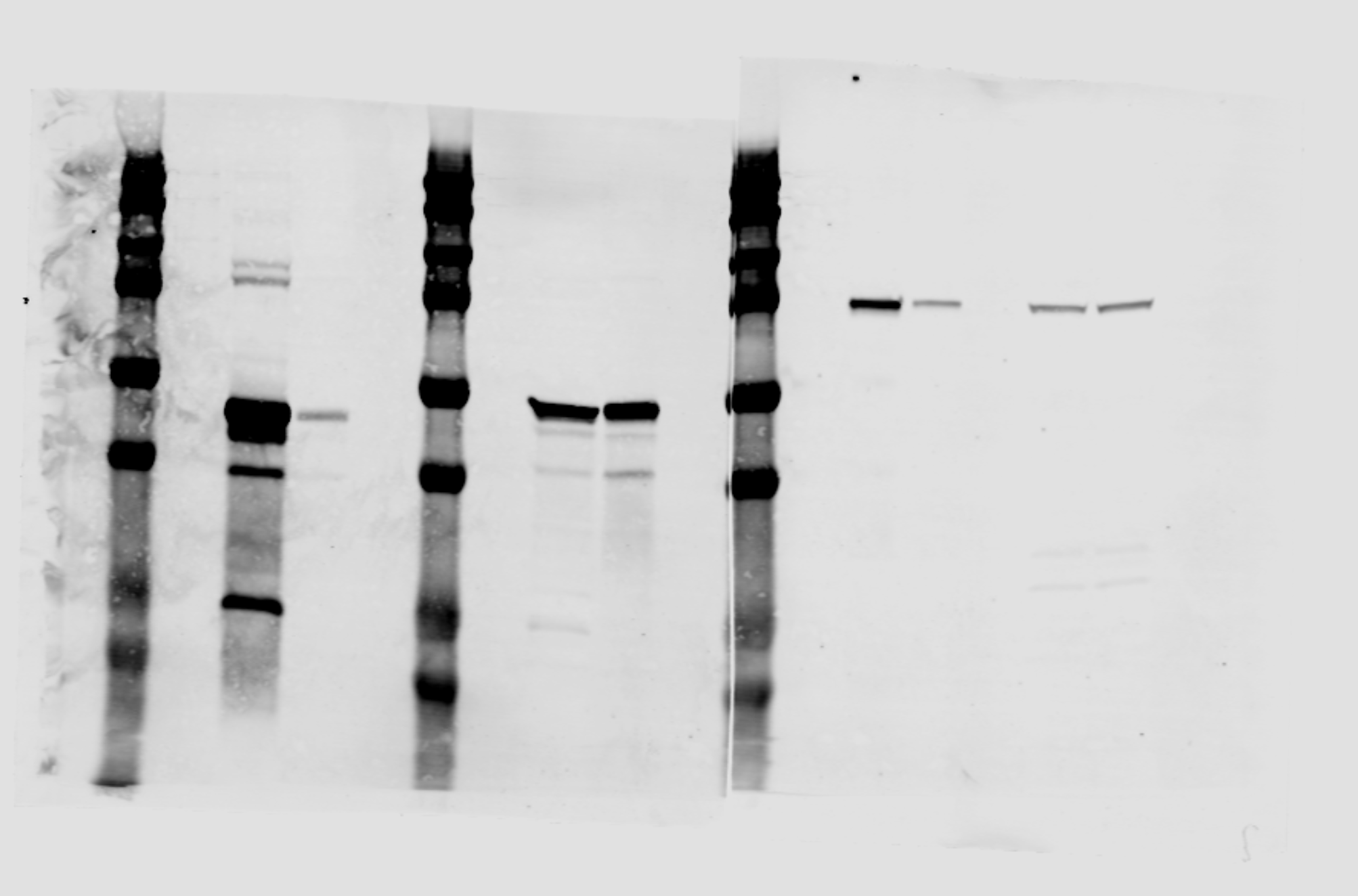

Supplement: Figure 7—source data 1. [file elife-80533-fig7-data1.zip › Fig 7sup1 MYC.png]

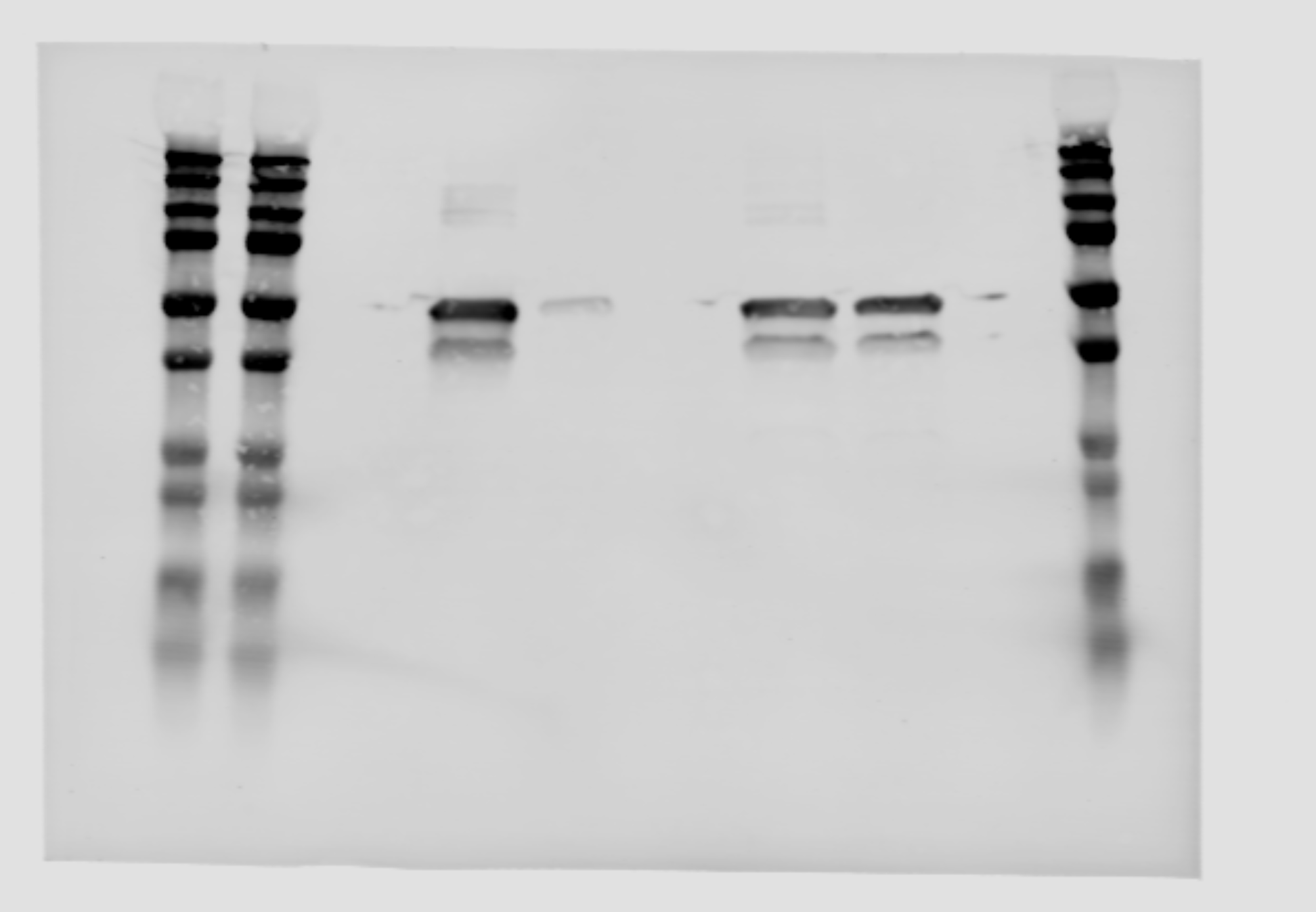

Supplement: Figure 7—source data 1. [file elife-80533-fig7-data1.zip › Fig 7sup1 GFP.png]

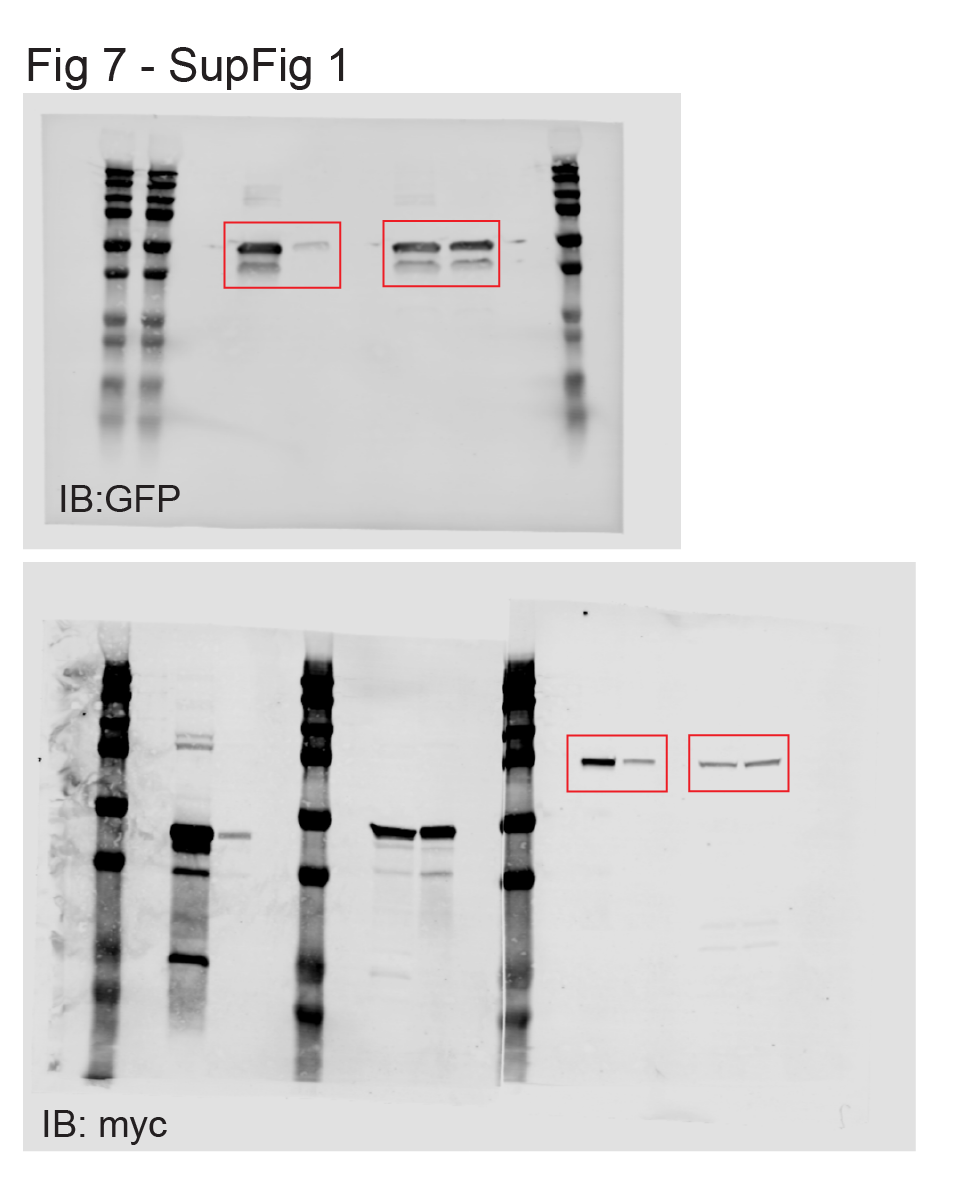

Supplement: Figure 7—source data 1. [file elife-80533-fig7-data1.zip › UncroppedWB_Fig 7.png]
