# Supplementary material for: Disrupting the ciliary gradient of active Arl3 affects rod photoreceptor nuclear migration
Source: eLife. 2023 Jan 4;12:e80533. doi: 10.7554/eLife.80533 (PMC9831603; doi:10.7554/eLife.80533)
Supplement: Supplementary file 1. [file elife-80533-supp1.docx]

**Supplementary Table 1: Primer sequences and usage.**

| **Primer Name** | **Sequence (from 5' to 3')** | **Use** |
| --- | --- | --- |
| AgeI-hArl3 | TATATATATAACCGGTGCCGCCACCATGGGCT | PCR Arl3 from pDEST47-Arl3 |
| hArl3-KpnI | GGCCCTAGGTACCGCTTTCTTCTTTGCATTG | PCR Arl3 from pDEST47-Arl3 |
| hArl3-T31N-F | ATAATGCTGGCAAGAaCACTCTTCTGAAGCA | PCR mutagenesis |
| hArl3-T31N-R | TGCTTCAGAAGAGTGtTCTTGCCAGCATTAT | PCR mutagenesis |
| hArl3-D67V-F | AACTGAATGTATGGGtCATTGGTGGACAGAG | PCR mutagenesis |
| hArl3-D67V-R | CTCTGTCCACCAATGaCCCATACATTCAGTT | PCR mutagenesis |
| hArl3-Q71L-F | GGGACATTGGTGGACtGAGGAAAATCAGACC | PCR mutagenesis |
| hArl3-Q71L-R | GGTCTGATTTTCCTCaGTCCACCAATGTCCC | PCR mutagenesis |
| hArl3-Y90C-F | GGAAGAATTATTTTGAAAATACCGATATTCTTATATgTGTAATCGACAGTGCAGACAGAAAAAGATTTGAAGA | PCR mutagenesis |
| hArl3-Y90C-R | TCTTCAAATCTTTTTCTGTCTGCACTGTCGATTACAcATATAAGAATATCGGTATTTTCAAAATAATTCTTCC | PCR mutagenesis |
| hArl3-D129N-F | CTCATCTTTGCTAATAAGCAGAATTTGCTCACAGCAGCCCCT | PCR mutagenesis |
| hArl3-D129N-R | AGGGGCTGCTGTGAGCAAATtCTGCTTATTAGCAAAGATGAG | PCR mutagenesis |
| hArl3-R149H-F | CTGCATACCATCCACGACCGAGTCTG | PCR mutagenesis |
| hArl3-R149H-R | CAGACTCGGTCGTGGATGGTATGCAG | PCR mutagenesis |
| hArl3-E164AD168A-F | TCAGCTCTCACAGGAGcGGGCGTTCAGGcTGGCATGAACTGGGTC | PCR mutagenesis |
| hArl3-E164AD168A-R | GACCCAGTTCATGCCAgCCTGAACGCCCgCTCCTGTGAGAGCTGA | PCR mutagenesis |
| ecT4dC-hPDE6D | GTGGTAGTCTTATGTCAGCCAAGGACGAG | PCR PDEδ from Dharmacon MHS6278-202829730 |
| hPDE6D-HindIII | GCGCGCAAGCTTTCATCAAACATAGAAAAG | PCR PDEδ from Dharmacon MHS6278-202829730 |
| GSThPDE6D-F | ctggttccgcgtggtagtattATGTCAGCCAAGGACGAG | PCR PDEδ from Dharmacon MHS6278-202829730 |
| GSThPDE6D-R | ggccgcaagcttcgtcatcatcaTCAAACATAGAAAAGTCTCACTCTG | PCR PDEδ from Dharmacon MHS6278-202829730 |
| AgeI-UNC119a-F | cccgggatccaccggtGCCGCCACCATGAAGGTGAAGAAAG | PCR UNC119A from Origene RC203758 |
| UNC119a-T2A-F | ATTCCTACAGTGGGACACCCAAAAAGCTTGAGGGCAGAGG | PCR UNC119A from Origene RC203758 |
| UNC119a-T2A-R | CCTCTGCCCTCAAGCTTTTTGGGTGTCCCACTGTAGGAAT | PCR UNC119A from Origene RC203758 |
| KpnI-UNC119-R | gtcgactggtaccgcGGGTGTCCCACTGTAG | PCR UNC119A from Origene RC203758 |
| hNPHP3-Seq-F1 | CTTCAAGTCCACTGGCTCGT | Sequencing human NPHP3 |
| hNPHP3-Seq-F2 | CAAGAGGGAGTTGGAGAGCA | Sequencing human NPHP3 |
| hNPHP3-Seq-F3 | GAAAAACCCTGAAGGAAAACC | Sequencing human NPHP3 |
| hNPHP3-Seq-F4 | CCAGCTCAGAGTCCTCCTTG | Sequencing human NPHP3 |
| hNPHP3-Seq-F5 | CTGCACTCTATCCGGGAGTC | Sequencing human NPHP3 |
| hNPHP3-Seq-F6 | ACAAAAGTGCAATGGCAACA | Sequencing human NPHP3 |
| hNPHP3-Seq-F7 | GATAATGCTCGGACCCTCAA | Sequencing human NPHP3 |
| hNPHP3-Seq-F8 | GCATCCTCGAGTTGGAGAAA | Sequencing human NPHP3 |
| hNPHP3-Seq-R1 | GCTTTTCCACCCAAGAGTGA | Sequencing human NPHP3 |
| hNPHP3-Seq-R2 | ACTCCTGGTTCTTGCTGACG | Sequencing human NPHP3 |
| AgeI-hNPHP3-F | GATCCACCGGTGCCGCCACCATGGGGACCGCCTCGTCGCT | PCR NPHP3 from GeneCopoeia GC-H2370 |
| hNPHP3-NotI-R | GGAGTGCGGCCGCCTACTTGTCATCGTCATCCTTGTAATCCCTTTGTCCTTGCTGAAGGA | PCR NPHP3 from GeneCopoeia GC-H2370 |
| hNPHP3-MYC-R1 | ATAAGCTTTTGTTCCCTTTGTCCTTGCTGAAGGA | Cloning myc tag onto NPHP3 |
| hNPHP3-MYC-R2-NotI | AGGAGTGCGGCCGCCTACAGATCCTCTTCTGAGATAAGCTTTTGTTCCCT | Cloning myc tag onto NPHP3 |
| mINPP5E-seqF2 | ttgcaagactcagtagccca | Sequencing mouse INPP5E |
| mINPP5E-seqF3 | gaattcaggagggctgctct | Sequencing mouse INPP5E |
| mINPP5E-seqF4 | cctcctacacagaccgagtc | Sequencing mouse INPP5E |
| mINPP5E-seqR1 | cagtgcttcctgtctctgaa | Sequencing mouse INPP5E |
| mINPP5E-seqR2 | atgaagaggaaggaggtgcc | Sequencing mouse INPP5E |
| mINPP5E-seqR3 | gaggctatgctgaggtccat | Sequencing mouse INPP5E |
| mINPP5E-NotI-R | AGGAGTGCGGCCGCTCAGGACACGGTGCAAACTG | PCR INPP5E from GFP-INPP5E |
| MYC-mINPP5E-F | ACAAAAACTCATCTCAGAAGAGGATCTGCCATCCAAGTCAGCTTGC | PCR INPP5E from GFP-INPP5E |
| mINPP5E-C644A-F | GCCAGAGCTCCAGTGCAGTTgcCACCGTGTCCTGAGCggccg | PCR mutagenesis |
| mINPP5E-C644A-R | cggccGCTCAGGACACGGTGgcAACTGCACTGGAGCTCTGGC | PCR mutagenesis |
| MYC-hRnd1-F | ACAAAAACTCATCTCAGAAGAGGATCTGTCCGGACTCAGATCC | PCR Rnd1 from Addgene 23227 |
| hRnd1-Not-R | aggagtgcggccGCTCACATAATGGAACA | PCR Rnd1 from Addgene 23227 |
| hRnd1-C229A-F | GAAAAGGCCAAAAGCgcTTCCATTATGTGAGC | PCR mutagenesis |
| hRnd1-C229A-R | GCTCACATAATGGAAgcGCTTTTGGCCTTTTC | PCR mutagenesis |
| AgeI-mArl13B | ggatccaccggtATGTTCAGTCTG | PCR mouse Arl13B |
| mArl13B-myc_link | CTCTTCTGAGATAAGCTTTTGTTCagcTGAGATCGTGTCCTGA | PCR mouse Arl13B |
| mArl13b-K216ER222E-F | GGAACAAGAGGAACGTGAGAGGGCTGAAGAAGTCCGGAAG | PCR mutagenesis |
| mArl13b-K216ER222E-R | CTTCCGGACTTCTTCAGCCCTCTCACGTTCCTCTTGTTCC | PCR mutagenesis |
| Age-kozak-HA-F | GGCCCGGGATCCACCGGTGCCGCCACCATGTACCCTTACGATGTACCGG | PCR RP2 from Dharmacon MMM1013-202842815 |
| HA-mRP2-F | ACCCTTACGATGTACCGGATTACGCAGGCTGCTGCTTCACTAAGAGGAG | PCR RP2 from Dharmacon MMM1013-202842815 |
| mRp2-Not-R | GAGTCGCGGCCGCTCATATCCCCATCTGGATCTC | Sequencing mouse RP2 |
| mRp2-Seq-F1 | GGAAGTCTTTTTGTGCTGTGC | Sequencing mouse RP2 |
| mRp2-Seq-R1 | AATTCCAAGGCAATCACAGG | Sequencing mouse RP2 |
